# Supplementary material for: Transcriptional Readthrough Interrupts Boundary Function in Drosophila
Source: Int J Mol Sci. 2023 Jul 12;24(14):11368. doi: 10.3390/ijms241411368 (PMC10379149; doi:10.3390/ijms241411368)
Supplement: Supplementary file 1 [file ijms-24-11368-s001.zip › ijms-2459890-supplementary.pdf]

Supporting information

SI Table S1. Primers for generating fragments

**Fragments**	**Primers**	**5’ - 3’**
**scs**	scs d	CGCTGCGAACTTCTCTTC
	scs r	CTGTATTCCTCAGTTATCGA
**scs^{min}**	scs m d	CGTCCGCATACGTCCG
	scs r	CTGTATTCCTCAGTTATCGA
**F7 HS3**	HS3_d	GTCGCAAGAACTTCACAACAG
	HS3_r	GCCATCATGGATGTGAAAGA
**PAS (sv40)**	sv40tr	GATACATTGATGAGTTTGG
	sv40td	GGATCTTTGTGAAGGAACCTTAC
**5’P**	1147	CATGATGAAATAACATAAGGTGGTC
	1152	GCTGCTGCTCTAAACGACG
**F7^{1+2+3}**	F7-1	GATTTCAAGCTGTGTGGCGGGGG
	F7-3	ATGTCGGCAATTCGGATTCCCGG
**F2 pHS2**	F2-47	TTTGTGAATCCGTACCC
	F2-48	TGAGCGAGTCCTTGAG
**F2**	F2D	GCTGAGGCGGCTGAGAAAG
	F2R	CAAGATACAATCAGCAAAGC
